# Identification of Functional Gene Modules Associated With STAT-Mediated Antiviral Responses to White Spot Syndrome Virus in Shrimp

**DOI:** 10.3389/fphys.2019.00212

**Published:** 2019-03-11

**Authors:** Guanghui Zhu, Shihao Li, Jun Wu, Fuhua Li, Xing-Ming Zhao

**Affiliations:** ^1^Institute of Science and Technology for Brain-Inspired Intelligence, Fudan University, Shanghai, China; ^2^Department of Computer Science and Technology, Tongji University, Shanghai, China; ^3^Laboratory for Marine Biology and Biotechnology, Qingdao National Laboratory for Marine Science and Technology, Qingdao, China; ^4^Institute of Oceanology, Chinese Academy of Sciences, Qingdao, China; ^5^Center for Ocean Mega-Science, Chinese Academy of Sciences, Qingdao, China

**Keywords:** antivirus response, co-expression network, gene module, STAT dsRNA, white spot syndrome virus, shrimp

## Abstract

White spot syndrome virus (WSSV) is one of the major threats to shrimp aquaculture. It has been found that the signal transducer and activator of transcription (STAT) protein plays an important role in the antiviral immunity of shrimp with a WSSV infection. However, the mechanism that underlies the STAT-mediated antiviral responses in shrimp, against WSSV infection, remains unclear. In this work, based on the gene expression profiles of shrimp with an injection of WSSV and STAT double strand RNA (dsRNA), we constructed a gene co-expression network for shrimp and identified the gene modules that are possibly responsible for STAT-mediated antiviral responses. These gene modules are found enriched in the regulation of the viral process, JAK-STAT cascade and the regulation of immune effector process pathways. The gene modules identified here provide insights into the molecular mechanism that underlies the STAT-mediated antiviral response of shrimp, against WSSV.

## Introduction

White spot syndrome virus (WSSV) is a highly lethal and contagious virus in penaeid shrimp, with huge economic consequences in commercial fishery and farming of the Pacific white shrimp, tiger prawn, Atlantic white shrimp, and so on. Once an outbreak of WSSV occurs, it wipes out entire populations in many shrimp farms within a few days and leads to enormous economic losses (Flegel et al., 2008). Due to the serious impact of WSSV on shrimp aquaculture, it is urgent to understand the molecular mechanisms that underlie WSSV pathogenesis in shrimp.

It has been reported that there are some genes that have revealed WSSV pathogenesis based on the transcriptome of shrimp with WSSV infection. For example, García et al. (2009) employed PCR technology to compare the transcriptomes in hemocytes of WSSV-infected shrimp with uninfected ones. They found that penaeidin-3 isoforms and crustin were over-expressed in hemocytes of WSSV-infected pre-challenged *Penaeus vannamei* (García et al., 2009). Shekhar et al. (2015) utilized DNA microarray technology to explore the genes of host immune responses to WSSV pathogenesis. They found some up- or down-regulated genes during WSSV infection in shrimp (Shekhar et al., 2015). Yu et al. (2017) found that a combination of single nucleotide polymorphisms in three genes (TRAF6, Cu/Zn SOD, and nLvALF2) were significantly associated with resistance to WSSV infection. The SNP loci in TRAF6, Cu/Zn SOD, and nLvALF2 were found to exhibit a significant effect on the resistance of shrimp to WSSV, while the expression of the three immune-related genes were affected by those SNPs (Yu et al., 2017). Li et al. (2013) used RNA-Seq technology to investigate the transcriptome of the shrimp between latent infection stage and acute infection stage. The genes that played an important role in host defense against WSSV and the genes that were possibly responsible for the rapid proliferation of WSSV, were identified (Li et al., 2013). Recently, the Toll, IMD, and JAK-STAT pathways were reported as the main pathways in the antiviral immunity against WSSV (Li and Xiang, 2013). It was found that the regulation of Toll and IMD pathways improved the anti-WSSV response in shrimp, while the silencing of the signal transducer and activator of transcription (STAT) gene, an important part of the JAK-STAT pathway, was proved effective both in reducing the WSSV-DNA copy number and the mortality of shrimp (Liu et al., 2007; Chen et al., 2008; Wen et al., 2014).

The above studies have provided a preliminary description of host responses against WSSV infection at the transcriptional level in shrimp, where the differentially expressed genes (DEGs) between WSSV-infected shrimp with and without the treatment of STAT. However, few of those DEGs are related to the antiviral response and the antiviral response genes may not be differentially expressed. This makes it difficult to understand the antiviral process mediated by the STAT gene. Biological networks can provide valuable information to better understand the mechanism of antiviral responses in a comprehensive and systematic way (Behura et al., 2011; Doering et al., 2012; Gupta et al., 2014; Li et al., 2014; Dai et al., 2017). In this work, based on the gene expression profiles of shrimp with an injection of WSSV and STAT double strand RNA (dsRNA), the gene co-expression network was constructed, where the network provided the functional relationships between genes. The gene modules, representing components consisting of densely connected genes in a co-expression network, were found to be suitable units to describe the metabolic disorders associated with WSSV infections. In the modules enriched with genes associated with STAT-mediated antiviral response against WSSV, infections were found related to biological processes such as the regulation of the viral process, modulation by host of symbiont transcription, JAK-STAT cascade, and the regulation of the immune effector process pathways. Furthermore, the network topology of these modules associated with the STAT-mediated antiviral response, provided clues to identify important genes and pathways in the antiviral response.

## Results and Discussion

**FIGURE 1:**
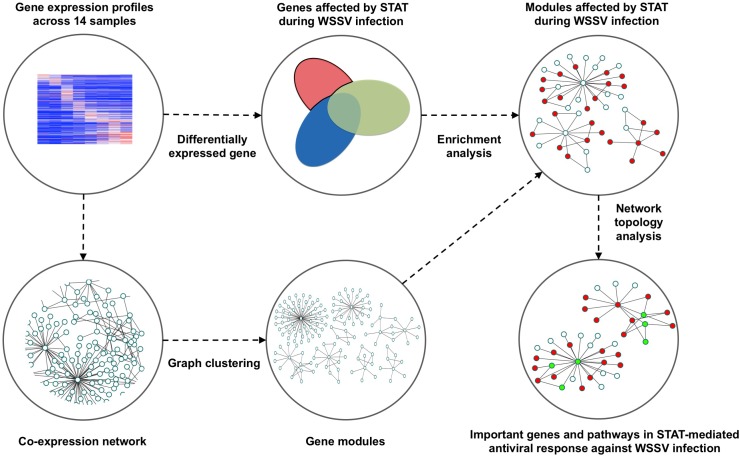
Pipeline to identify the gene modules and important genes that play an important role in the STAT-affected antiviral response during the infection of WSSV.

The schematic for the analysis pipeline is shown in Figure 1. The gene co-expression network was constructed based on gene expression profiles across 14 shrimp samples injected with STAT dsRNA and WSSV (Table 1). The densely connected components, i.e., gene modules, of the network were further detected as functional units in the antiviral process. These modules were found enriched with metabolic functions, previously reported as dysfunctional in WSSV-infected shrimps. Furthermore, the information of network topology of those modules affected by STAT during WSSV infection, was utilized to identify important genes and pathways in the antiviral response affected by STAT against WSSV infection.

**Table 1:** The 14 samples used to generate gene expression profiles.

Sample ID	Time after injection and the injected material
E0	48 h after injection of EGFP dsRNA
S0	48 h after injection of STAT dsRNA
EP24	72 h after injection of EGFP dsRNA, 24 h after injection of PBS
EP48	96 h after injection of EGFP dsRNA, 48 h after injection of PBS
EP72	120 h after injection of EGFP dsRNA, 72 h after injection of PBS
EW24	72 h after injection of EGFP dsRNA, 24 h after injection of WSSV
EW48	96 h after injection of EGFP dsRNA, 48 h after injection of WSSV
EW72	120 h after injection of EGFP dsRNA, 72 h after injection of WSSV
SW24	72 h after injection of STAT dsRNA, 24 h after injection of WSSV
SW48	96 h after injection of STAT dsRNA, 48 h after injection of WSSV
SW72	120 h after injection of STAT dsRNA, 72 h after injection of WSSV
SP24	72 h after injection of STAT dsRNA, 24 h after injection of PBS
SP48	96 h after injection of STAT dsRNA, 48 h after injection of PBS
SP72	120 h after injection of STAT dsRNA, 72 h after injection of PBS


### Identification of Gene Modules Affected by WSSV Infection and STAT dsRNA

**FIGURE 2:**
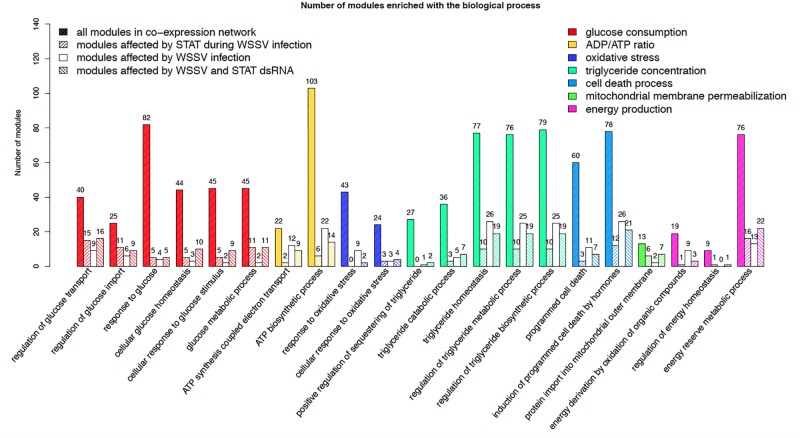
Number of all the modules in the co-expression network, modules affected by STAT during WSSV infection, modules affected by WSSV infection, and modules affected by WSSV and STAT dsRNA, significantly enriched with the processes relevant to the metabolic disorders previously reported in WSSV-infected shrimp.

To identify the gene modules affected by STAT during WSSV response, the gene co-expression network was constructed based on the gene expression across 14 samples injected with STAT dsRNA and WSSV. Only genes with top a 30% variance of the gene expression were used to construct the network. 0.01 was chosen as the *p*-value threshold for the Pearson correlation coefficient (PCC) between two genes, to further screen out the edges of the network. The co-expression network consisted of 15,167 genes and 2,288,537 edges, representing significant correlations between these genes. In total, 1873 modules with default parameters were detected by the ClusterOne algorithm (Nepusz et al., 2012). To investigate the role of these modules in the antiviral process, the functional enrichment analysis was performed to identify the biological processes that were significantly enriched in every module. In particular, the two-tailed Fisher’s exact test with a *p*-value threshold of 0.05 was used to identify processes that were significantly enriched in each module. Referring to the report from a previous study on the metabolic changes in WSSV-infected shrimp, the metabolic system of WSSV-infected shrimp was mainly changed in glucose consumption, plasma lactate concentration, activity of glucose-6-phosphate dehydrogenase, ADP/ATP ratio, oxidative stress, triglyceride concentration, the cell death process, mitochondrial membrane permeabilization, energy production, and upregulation of the voltage-dependent anion channel (Chen et al., 2011). The identified modules were significantly enriched within biological processes relevant to almost all of these reported abnormalities. The number of modules enriched with the processes relevant to these reported disorders is shown in Figure 2. Additionally, there were 12 modules enriched with the biological processes of regulation of the JAK-STAT cascade and 13 modules enriched with modulation by the host of symbiont transcription. Considering the above, these modules can be used as a signature to characterize the influence of WSSV infection and STAT dsRNA interference on the metabolism system.

### Identification of Genes Affected by STAT During WSSV Infection

To find network modules affected by STAT during WSSV infection, the DEGs were identified based on gene expression profiles across the 14 samples. Three sets of DEGs were obtained by comparing three groups of samples. The WSSV-infected samples (EW_48 and EW72) were compared with controls (EP48 and EP72) to identify DEGs affected by WSSV infection. The samples injected with STAT dsRNA (SP48 and SP72) were compared with the controls (EP48 and EP72) to identify DEGs affected by STAT. The samples injected with both WSSV and STAT dsRNA (SW48 and SW72) were compared with the controls (EP48 and EP72) to identify DEGs affected by both WSSV and STAT dsRNA. A functional enrichment analysis was performed on each set of DEGs, to evaluate whether these gene sets were associated with the regulation of STAT and infection of WSSV. As shown in Table 2, the 3144 genes affected by WSSV infection were significantly enriched with biological processes such as the positive regulation of JUN kinase activity, natural killer cell activation, B cell activation, T cell activation, and the regulation of an adaptive immune response. Other than the four processes associated with activity of immunocytes, JUN kinase activity has been reported to promote viral replication and plays a role in WSSV infection in shrimp (Shi et al., 2012; Wang et al., 2017). The 2685 genes affected by STAT dsRNA were significantly enriched with processes such as hemocyte differentiation, embryonic hindlimb morphogenesis, positive regulation of an inflammatory response, and the viral process. These processes have been reported to be associated with STAT, indicating that the STAT dsRNA interference indeed affected the cascades of STAT signaling in the STAT dsRNA-injected samples (Luo and Dearolf, 2001; Grebien et al., 2008; Kiu and Nicholson, 2012). The 2785 genes affected by both WSSV and STAT dsRNA were significantly enriched with processes such as the negative regulation of JAK-STAT cascade, cell death, the viral process, and the oxidation-reduction process. There were accidental factors in the identified genes affected by both WSSV and STAT dsRNA, such as technical noise and genes that were affected only by either WSSV or STAT dsRNA. To further exclude accidental factors, the genes specially affected by STAT during WSSV infection were obtained as the remaining part of the genes affected by both WSSV and STAT dsRNA, after removing the genes in the gene set associated with WSSV infection and the gene set affected by STAT interference (Supplementary File S1). As shown in the infection, 1723 genes particularly affected by STAT during WSSV infection were significantly enriched with the negative regulation of the JAK-STAT cascade, lymphocyte differentiation, and positive regulation of immune effector process. It suggested that the part of the genes annotated with lymphocyte differentiation and immune effector process were particularly regulated by the mutual effect of STAT dsRNA interference and WSSV infection, rather than individually regulated by the WSSV or STAT dsRNA alone. The genes particularly affected by STAT during WSSV infection were also significantly enriched with the regulation of an adaptive immune response. While shrimp are generally assumed to have no adaptive immune response, recent studies have shown that shrimp can obtain immune responses to specific pathogens including bacterium and viruses (Yang et al., 2012; Lin et al., 2013).

**Table 2:** The biological processes significantly enriched in the genes affected by WSSV infection, genes affected by STAT, genes affected by both WSSV and STAT dsRNA, and genes specially affected by STAT during WSSV infection.

The genes affected by WSSV infection	The genes affected by STAT	The genes affected by both WSSV and STAT dsRNA	The genes specially affected by STAT during WSSV infection
Positive regulation of JUN kinase activity (0.00495)	Embryonic hindlimb morphogenesis (0.000952)	Negative regulation of JAK-STAT cascade (0.00627)	Regulation of adaptive immune response based on somatic recombination of immune receptors built from immunoglobulin superfamily domains (0.0229)
Natural killer cell activation (0.0126)	Hemocyte differentiation (0.0354)	Viral process (0.011)	Negative regulation of JAK-STAT cascade (0.0246)
Positive regulation of B cell activation (0.0361)	Interspecies interaction between organisms (0.00255)	Cell death (0.0174)	Regulation of adaptive immune response (0.027)
Immunoglobulin V(D)J recombination (0.0379)	Positive regulation of inflammatory response (0.0343)	Somatic diversification of immune receptors (0.0451)	Lymphocyte differentiation (0.046)
T cell activation involved in immune response (0.041)	Viral process (0.00354)	Oxidation-reduction process (0.00144)	Positive regulation of immune effector process (0.0463)


*The *p*-values obtained through two-tail Fisher’s exact test were represented in the brackets.*

### Identification of Modules Specially Affected by STAT During WSSV Infection

**FIGURE 3:**
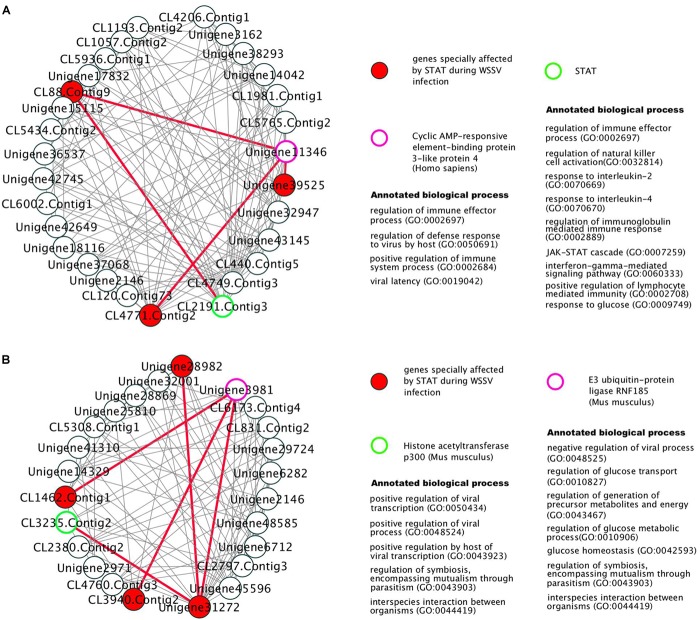
The gene co-expression network in two modules, module_233 **(A)** and module_1253 **(B)**, particularly affected by STAT during WSSV infection. The edges connecting important genes to STAT-WSSV-jointly affected genes were marked as red thick lines.

The 381 modules significantly enriched with genes particularly affected by STAT during WSSV infection, were identified as the candidate modules particularly affected by STAT during WSSV infection (CMASWs) (Supplementary File S2). These CMASWs were enriched with all the biological processes relevant to the metabolic changes in WSSV-infected shrimp (Figure 2). To obtain a comprehensive view of the metabolic disorders affected by WSSV and STAT dsRNA, the number of CMASWs significantly enriched with every relevant biological process was compared with that of candidate modules affected by WSSV and the modules affected by both WSSV and STAT. The 531 modules enriched with genes affected by WSSV and 574 modules enriched with genes affected by both WSSV and STAT were identified as candidate modules affected by WSSV and affected by both WSSV and STAT, respectively (Supplementary Files S3, S4). The number of CMASWs enriched with every process relevant to glucose consumption was more than that of candidate modules affected by WSSV, suggesting that glucose consumption was the potential aspect of the metabolic system affected by STAT dsRNA interference in WSSV-infected shrimps. The number of CMASWs significantly enriched with every process related with ATP synthesis, triglyceride concentration, and the cell death process was less than that of candidate modules affected by WSSV. In particular, the number of candidate modules affected by both WSSV and STAT, enriched with every process related with ATP synthesis and cell death, was also less than that of candidate modules affected by WSSV, indicating that these two aspects of the metabolic disorder were alleviated by STAT dsRNA interference. Additionally, there were nine CMASWs significantly enriched with the regulation of the JAK-STAT cascade biological process, 13 CMASWs enriched with the regulation of the viral process, and 24 CMASWs enriched with the regulation of the immune response, suggesting that these CMASWs were indeed associated with the antiviral response affected by STAT against WSSV. Considering that the densely connected genes in a CMASW tended to achieve functions together, the topology of a CMASW provided clues to identify important genes in the STAT-affected antiviral response against WSSV. As shown in the Figure 3A, the gene Unigene11346 in the CMASW module_233 was not differentially expressed between shrimp injected with both STAT dsRNA and WSSV (samples SW48 and SW72) and the control (samples EP48 and EP72), but the gene Unigene11346 was associated with three genes, CL88.Contig9, Unigene39525, and CL4771.Contig2, that were particularly affected by STAT during WSSV infection in the module. Despite this, Unigene11346 was not differentially expressed during the STAT-mediated antiviral response. Its relevant functions, such as the regulation of the immune effector process, regulation of the defense response to a virus by the host, and the positive regulation of the immune system process, were potentially regulated by the three associated DEGs. The CL2191.Contig3 (STAT) was a hub gene connected with 13 (44.8%) out of 29 genes in the module, suggesting that STAT had significant influence on this module. While STAT was annotated with the immune effector process, positive regulation of the immune system process, regulation of the immunoglobulin mediated immune response, natural killer cell activation, and the response to interleukins, other genes in the modules were potentially involved in these processes. The other gene CL4749.Contig3 was associated with 23 (79.3%) genes including all three genes particularly affected by STAT during WSSV infection and the other two important genes, Unigene11346 and CL2191.Contig3, suggesting the important role of CL4749.Contig3 in the STAT-mediated antiviral response. As shown in Figure 3B, the module_1253 was associated with a modification by the host of symbiont morphology or physiology, modulation by host of viral transcription, regulation of viral transcription, and interspecies interaction between organisms. Both the genes Unigene3981 and CL3235.Contig2 were annotated with these biological processes. While these two genes were not identified as DEGs, the three associated genes, CL1462.Contig1, CL3940.Contig2, and Unigene31272, that were identified as genes particularly affected by STAT during WSSV infection, tended to participate in these processes. Among these three genes particularly affected by STAT during WSSV infection, Unigene31272 was a hub gene connecting 17 (70.8%) genes out of 24 in the module, indicating they played an important role in the antiviral process. Unigene3981 was also annotated with the regulation of the glucose metabolic process, glucose homeostasis, and the regulation of the glucose metabolic process, which are relevant to the disordered aspects in the metabolic system in WSSV-infected shrimp.

## Conclusion

Aiming to better understand the antiviral responses affected by STAT against WSSV in shrimp, the gene modules relevant to the antiviral responses mediated by STAT were identified from the gene co-expression network. These modules were found to be associated with the biological processes that underlie the metabolic changes during WSSV infection. The important genes and antiviral responses were further identified based on the network topology of these modules. For example, Unigene11346, CL4749.Contig3, Unigene3981, and CL3235.Contig2 were identified as important genes in the antiviral response. Functional enrichment analysis suggests that these genes are enriched with biological processes that potentially underlie antiviral responses, e.g., the immune effector process, regulation of the defense response to a virus by the host, regulation of the immune system process, modification by the host of symbiont morphology or physiology, modulation by the host of viral transcription, regulation of the viral transcription, and the interspecies interaction between organisms. These findings provide insights into the molecular mechanisms that underlie shrimp antiviral responses.

## Materials and Methods

### Shrimp Maintenance and Virus Preparation

Healthy shrimp *Litopenaeus vannamei*, with a body weight of 4.4 ± 1.0 g were acquired from an aquaculture farm and reared in the lab. They were maintained in natural, aerated seawater at 25 ± 1°C and fed with commercial feed twice a day. WSSV virions were purified from the tissues of infected shrimp, following the method reported by Sun et al. (2013) and the stock solution was 3.5 × 10^4^ copies/μL in PBS.

### Preparation of Samples

Synthesis and optimization of the dose of the dsRNA of the target gene *LvSTAT* (ds*LvSTAT*) for gene silencing, fully followed the methods reported by Wen et al. (2014). Ten micrograms ds*LvSTAT* was injected into each shrimp for gene silencing. At the same time, a fragment of enhanced green fluorescent protein (EGFP) gene from the pEGFP-N1 plasmid was used for synthesis of control dsRNA (dsEGFP) following the same method (Wen et al., 2014).

Four groups including SP, EP, SW, and EW were set in the following experiments. Each group contained 20 shrimps. In SP and EP, each shrimp was injected with 10 μg ds*LvSTAT* and 10 μg dsEGFP, respectively, and then each shrimp was injected with 10 μL PBS 48 h later. In SW and EW, each shrimp was injected with 10 μg ds*LvSTAT* and 10 μg dsEGFP, respectively, and then each shrimp was injected with 10 μL WSSV solution containing 8.0 × 10^3^ copies, 48 h later. The time of PBS or WSSV injection was recorded as 0 h. Before PBS or WSSV injection, 48-h post ds*LvSTAT* or dsEGFP injection, cephalothorax samples of three shrimp from each group were collected separately for RNA extraction. They were named as S0 and E0. At 24, 48, and 72-h post WSSV or PBS injection, cephalothorax samples of three shrimp from each group were collected separately for RNA extraction. These samples were named EP24, SP24, EW24, SW24, EP48, SP48, EW48, SW48, EP72, SP72, EW72, and SW72, respectively.

### Illumina Sequencing

Paired-end RNA sequencing was performed to generate the transcriptome for each sample. In brief, the total RNA of each sample was extracted with a RNAisol reagent (Takara, Japan) and treated with DNase I. The RNA amounts were estimated spectrophotometrically by a NanoDrop 2000 spectrophotometer (Thermo Fisher Scientific, United States). Polyadenylated (polyA+) RNA was purified from the total RNA using Sera-mag oligo(dT) beads, fragmented to a length of 100–500 bases, reverse transcribed using random hexamers, and end repaired and adaptor-ligated according to the manufacturer’s protocol (Illumina). Ligated products of 300–500 bp were excised from agarose and PCR-amplified (15 cycles). Products were cleaned using a MinElute column (Qiagen) and single-end sequenced on a Genome Analyzer II (Illumina), according to manufacturer’s instructions. The raw sequencing data has been deposited in the Sequence Read Archive (SRA) database (SRA accession: SRP159438).

### Preprocessing of RNA-Seq Data

The reference assembly from a previous work was used as the reference transcriptome (Wei et al., 2014). More detailed information about the sequencing data can be found in Supplementary File S5. The reads from each sample were mapped to the reference assembly with RSEM software (Li and Dewey, 2011). The percentage of total mapped reads ranged from 78.34 to 85.40% across the 14 samples (Supplementary File S6). The expression of each gene was calculated as the RPKM with HTSeq software (Anders et al., 2015). After the gene expression values were log2-transformed and normalized with the quantile function from the Limma R package, the gene expression were further normalized with the median subtracted within each sample (Gentleman et al., 2004).

### Identification of Differentially Expressed Genes

The genes that were differentially expressed between distinctive conditions were identified as those genes whose expression had changed more than fourfold. Specifically, only samples affected by WSSV infection and STAT dsRNA interference at both 48 and 72 h post-infection (hpi) were considered here, since the copy number of WSSV was reported with no difference between shrimps injected with STAT dsRNA at 12 hpi and the control (Wen et al., 2014). For each time point, a set of DEGs were identified between two different conditions, and the intersection of the two sets of DEGs obtained at two time points were used for further analysis. In detail, the samples obtained after injection of WSSV (EW_48 and EW72) were compared with the controls (EP48 and EP72) to identify the genes affected by WSSV infection, and the same for samples injected with STAT dsRNA (SP48 and SP72) and samples injected with both WSSV and STAT dsRNA (SW48 and SW72) (Supplementary Files S7, S8).

### Construction of Gene Co-expression Network

For the gene co-expression network, the PCC was calculated to quantify the association between each pair of genes. Only genes with the top 30% variance of gene expression across samples were included for further analysis. To keep only significant correlations between genes, and to further reduce the noise in the network, each association was required to have a *p*-value no larger than 0.01. As a result, the degree distribution of the gene co-expression network followed a power-law distribution with the parameter alpha equal to 2.12, consistent with a previous conclusion that biological networks were scale-free networks (Albert, 2005; Clauset et al., 2009).

### Identification of Modules From Gene Co-expression Network

The modules in the gene co-expression network were detected by ClusterOne, which is a popular tool widely used in the bioinformatics field, where the default parameter was employed for ClusterOne (Nepusz et al., 2012; Giovannetti et al., 2014; Fuchsberger et al., 2016; Wojtuszkiewicz et al., 2016; Yang et al., 2016). The gene components of each module can be found in Supplementary File S9. The modules significantly enriched with DEGs were detected with a two-tail Fisher’s exact test with a *p*-value smaller than 0.05, and the same for the detection of biological processes enriched in each module. The functional annotation of shrimp genes was obtained with the functional annotation transferred from their homologous genes. The shrimp gene sequences were queried against the NCBI non-redundant protein sequence database, the NCBI nucleotide sequence database and the EggNOG database with BLAST, where a gene was regarded as a homologous gene with an *E*-value smaller than 1e-5 (Huerta-Cepas et al., 2015). Consequently, 665,531 biological processes were annotated to 12,505 shrimp genes (available in Supplementary File S10).

## Data Availability

The datasets generated for this study can be found in sequence read archive (SRA), SRP159438.

## Author Contributions

FL and X-MZ conceived the study and designed the experiments. SL performed the experiments. GZ, JW, and X-MZ analyzed and interpreted the data. GZ and SL wrote the manuscript. SL, GZ, JW, FL, and X-MZ discussed the work and revised the manuscript.

## Conflict of Interest Statement

The authors declare that the research was conducted in the absence of any commercial or financial relationships that could be construed as a potential conflict of interest.

## Supplementary Material

FILE S1 Genes specially affected by STAT during WSSV infection.

FILE S2 Candidate modules specially affected by STAT during WSSV infection.

FILE S3 Gene modules affected by WSSV.

FILE S4 Modules affected by both WSSV and STAT.

FILE S5 Gene sequence.

FILE S6 Qualities of RNA sequencing of samples.

FILE S7 Genes affected by WSSV infection.

FILE S8 Genes affected by both WSSV and STAT dsRNA.

FILE S9 All gene modules.

FILE S10 GO annotation.
